# Instrument fracture in periodontal therapy: ethical disclosure and clinical management - a case report

**DOI:** 10.1186/s12903-024-04349-9

**Published:** 2024-05-21

**Authors:** Hassan Yaqoob, Ali Sadiq, Muhammad Karim, Syed Murtaza Raza Kazmi

**Affiliations:** Dental Section, Department of Surgery, The Aga Khan University, Karachi, Pakistan

**Keywords:** Periodontal instrument fractures, Sickle scaler, Ethics, Management, Retrieval, Instrument replacement, Treatment protocol

## Abstract

Periodontal instrument fractures are rare events in dentistry, with limited literature available on their occurrence and management. This case report highlights an incident involving the fracture of a periodontal sickle scaler blade during manual instrumentation for the removal of calculus. The fracture occurred during instrumentation on the mesial surface of the maxillary right second molar, and the separated blade was subsequently pushed into the sulcus. A radiographic assessment was performed to verify the precise location of the fractured segment. Following confirmation, the broken blade was subsequently retrieved using curved artery forceps. The case report highlights factors contributing to instrument fractures, emphasizing the importance of instrument maintenance, sterilization cycles, and operator technique. Ethical considerations regarding patient disclosure, informed consent, and instrument retrieval methods are well discussed. This case underscores the importance of truthful communication, the proper use of instruments, equipment maintenance in dentistry, and the significance of ongoing professional development to enhance treatment safety, proficiency, and ethical standards in dental care.

## Introduction

**Fig. 1:**
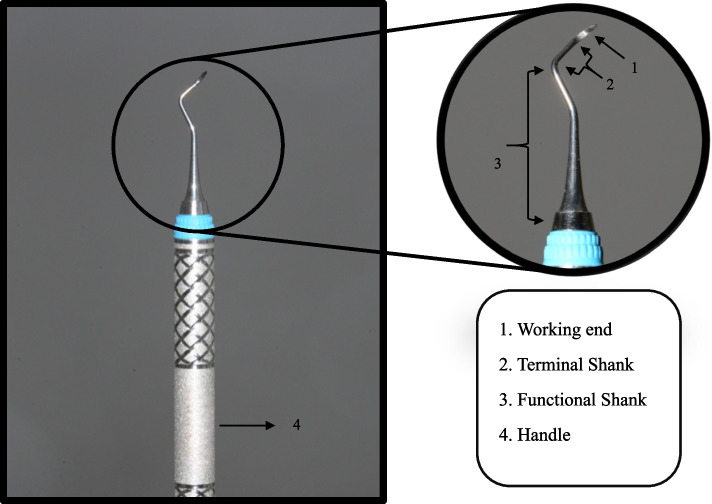
Sickle scaler. See the zoom-in section for detailed view of instrument parts

Dental plaque constitutes a complex biofilm, colonised by various species of bacteria, and serves as a primary causative factor for periodontitis [1]. If plaque is left undisturbed, it mineralizes and forms calculus, which further facilitates plaque accumulation [2]. The manual instruments such as curettes and sickles are commonly used for debridement, which requires regular sharpening for optimum results [3]. However, this can compromise the integrity of the working ends of the sickle scaler (Fig. 1) and curette, rendering them more susceptible to fractures and reducing their longevity [4, 5]. Increased sterilization cycles may also weaken these instruments, increasing the likelihood of their fracture [5]. Additionally, the operator’s proficiency and inferior metallurgy of the instruments are vital in predisposing instrument fractures [2]. 

Periodontal instrument fractures are extremely rare events [6]. The existing literature is notably deficient in guiding the management of such incidents, with only two case reports available as per authors knowledge [6, 7]. When dealing with an instrument fracture, it is crucial to weigh the risks and benefits associated with either removing or retaining the broken fragment of the instrument. The removal of the instrument has the potential for injury to delicate neurovascular structures such as inferior alveolar nerves and arteries, as well as the nasal cavity and maxillary sinus, while leaving it may cause persistent pain, abscess, or secondary infection [8, 9]. The patient must be fully informed in any such situation, and the advantages and disadvantages of either removing or leaving the fragment in place must be thoroughly explained and meticulously considered before making a decision [10]. 

This report highlights the effective patient management and retrieval of a broken periodontal sickle scaler from a deep periodontal pocket. Additionally, it delineates the protocol employed for instrument retrieval and provides recommendations regarding instrument replacement.

## Case report

A 64-year-old male patient presented at the dental clinics of a tertiary care hospital with a complaint of food impaction and widespread staining on teeth. Additionally, he sought consultation regarding the prosthetic replacement of tooth #25. The patient’s medical history comprised of chronic obstructive pulmonary disease (COPD), stroke, and ulcerative colitis, for which he was receiving medication. The patient had a habit of smoking cigarettes frequently, along with the use of alcohol and recreational drugs. Intraoral examination indicated compromised oral hygiene, as evidenced by the presence of plaque, supra and sub gingival calculus deposits, and deep periodontal pockets.

### Intra-oral examination and radiographic evaluation

**Fig. 2:**
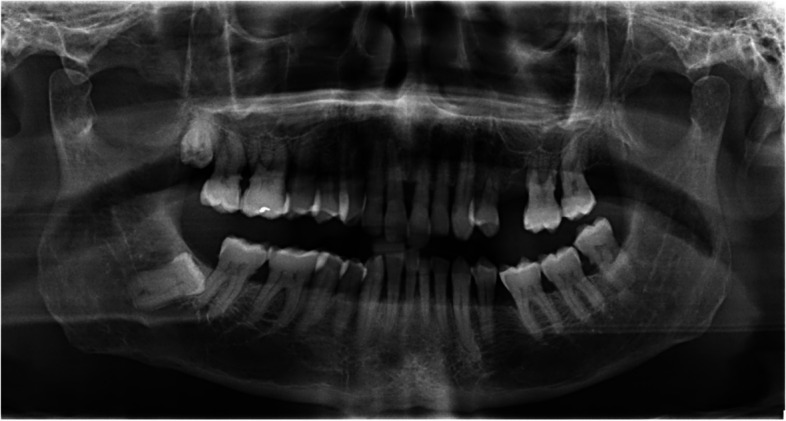
Orthopantomogram (OPG)

**Fig. 3:**
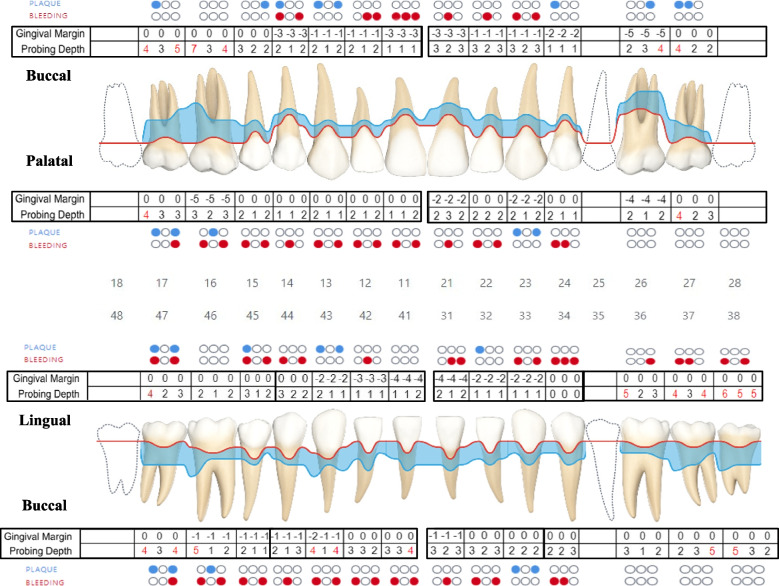
Six Point Pocket Charting. Probing depth (  ) Gingival recession (  )

Prior to commencing the treatment, a pre-operative orthopantomogram (x-ray) was taken (Fig. 2). The Basic Periodontal Examination (BPE) Index score was documented, indicating a code of 4 in the first and fourth sextants, and a code of 3 in the third, fifth, and sixth sextants. This led us to record a 6-point pocket charting (Fig. 3) for the patient’s entire dentition according to the recommended treatment guidelines of BPE [11]. The patient was diagnosed to have generalized periodontitis stage III, grade C, currently unstable with smoking as a risk factor [12]. The patient was informed, and consent was obtained regarding non-surgical periodontal therapy as the first step of treatment.

### Case

The patient had periodontal pockets with moderate to severe probing depth. The recommended approach for individuals with such conditions entails the removal of supragingival biofilm and calculus, coupled with subgingival instrumentation [13]. Thus, an ultrasonic scaler was employed to eliminate supragingival calculus. Subsequently, manual instruments such as curettes and sickle scalers were used to remove sub-gingival calculus under local anesthesia that have been reported to be effective in reducing probing pocket depth of ≥ 4 mm [14]. Nevertheless, the procedure was disrupted when the blade of the sickle scaler unexpectedly fractured during instrumentation on the mesial surface of the maxillary right second molar and consequently was pushed into the sulcus.

### Management

**Fig. 4:**
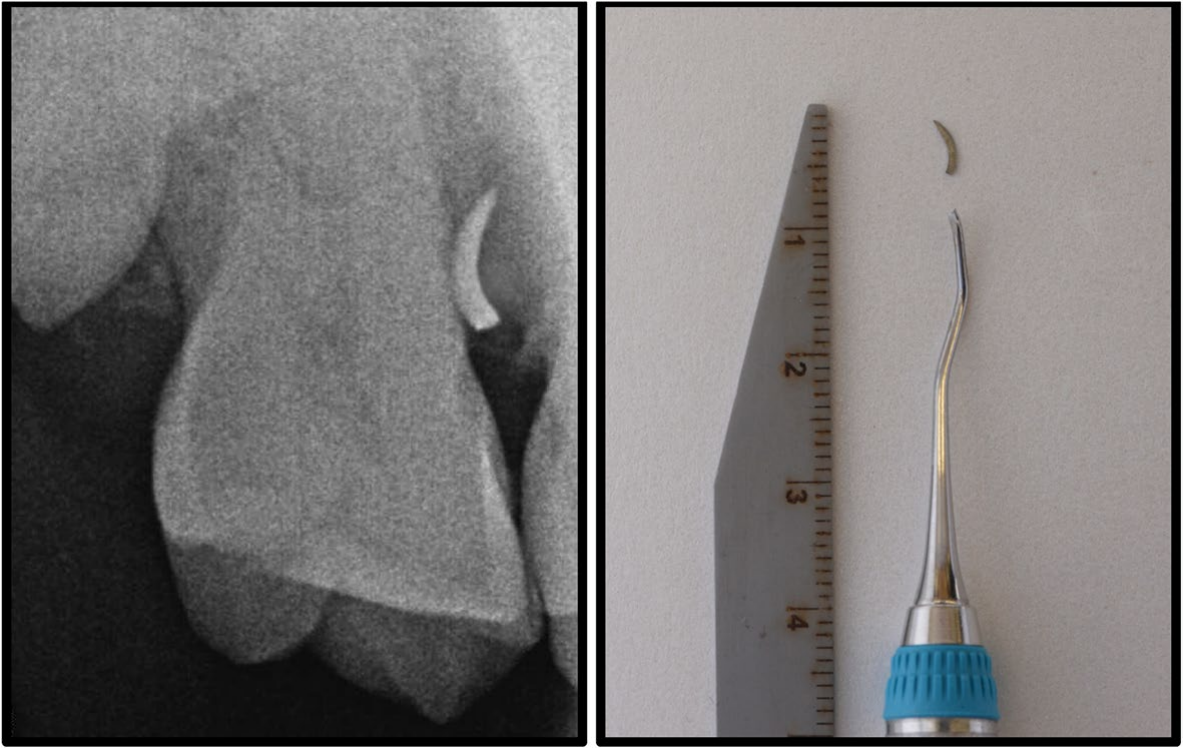
(A) Fractured Blade of sickle scaler. Note the location which is in the middle 1/3rd of root. (B) Clinical picture taken just after the removal of blade

Upon instrument fracturing, the procedure was immediately halted, and the patient was positioned upright to prevent potential aspiration or ingestion of the fractured fragment. The patient was informed regarding the incident, along with the associated risks and benefits of removing and leaving the broken part in-situ. He was instructed to keep his mouth open and refrain from swallowing to prevent additional complications. The fracture segment was promptly searched within the oral cavity to verify its location—whether it remained inside or had been removed during the suctioning process. A simultaneously done site-specific periapical radiograph confirmed the presence and location of the fractured instrument segment (Fig. 4a). This was followed by creating a pathway for direct visualization of the fracture component by deflecting the gingiva with the assistance of a dental UNC probe. Subsequently, the fragment was retrieved using curved artery forceps (Fig. 4b).

## Discussion

Instrument fracture inside the oral cavity is a frustrating and undesirable occurrence [15]. There is limited data available regarding periodontal instrument fractures [6, 7]. These fractures may result from procedural errors, such as excessive force application without a stable fulcrum, use of aged instruments subjected to sterilization and/or sharpening cycles, or compromised metallurgy [5, 7, 16]. On average, a curette fractures approximately after 14.34 sterilization cycles [2]. Lieu et al. demonstrated that the size of the instrument’s working end decreases when scaling cycles increase, elevating the fracture risk during instrumentation [2]. Furthermore, sharpening contributes to discernible wear of the instruments, which can lead to breakage, as reported by Lieu et al. where the #11/12 Gracey curette fractured when the blade width reduced to less than 0.55 mm [2]. This case report raises a similar incidence wherein the fractured blade exhibited a width of 0.4 mm when measured after the event.

Kwon et al. [17] explored the correlation of instrument fracture with type of procedure, breakage point, and operator expertise. Root planning exhibited the highest fracture rate (63.8%), amounting to around 16 fractures per one thousand curettes utilized. The upper one-third of the blade was the most frequent site of fracture, accounting for 44.8% of fractures, followed by the terminal shank (29.3%). Additionally, the authors reported that the fracture incidents were independent of the clinical expertise of the operator. Our case reflected a similar incidence of fracture site at the upper one-third of the blade despite maintaining stable fulcrum by the practicioner [17].

Instrument quality and maintenance, therefore, prove imperative in averting such incidents. Periodontal hand instruments are essentially made up of martensitic steel, which is well known for its durability and resistance to corrosion [18]. Tal et al. found high carbon steel (HCS) dental curettes more wear-resistant than stainless steel (SS) ones [19]. However, the widespread use of stainless steel instruments persists due to their biocompatibility, adherence to international standards (ISO7153-1), and longevity when properly maintained [7]. Innovations in instrument coatings, like multilayered filtered arc coatings, extend their lifespan and clinical utility, with some instruments retaining their clinical usefulness for up to 11 months [20]. Nonetheless, ensuring optimum instrument quality requires meticulous compliance with maintenance guidelines by clinicians. This includes conducting regular inspections before packaging to preemptively address wear and/or fatigue caused by repeated use of the instrument [6]. 

Fractured instrument segments entail risks of swallowing or aspiration, persistent pain, abscess formation, septicemia, or bleeding [21]. Timely removal of the fragment is essential to prevent migration into adjacent spaces; for instance, a fragment fractured on the lingual side of the mandible can migrate to the submandibular or parapharyngeal space. This may potentially cause catastrophic complications, such as laceration of the maxillary artery or jugular vein [22, 23]. Delayed removal increases the risk of infection and tissue destruction due to inflammation, thrombosis, erosion into the carotid artery or its branches, and nerve interference [24]. Hence, it is imperative to immediately search for and retrieve the fractured fragment [25], as was done in this case by maintaining a composed and calm demeanour.

In healthcare, it is crucial to promptly identify the errors and inform the patient about the potential injury [26]. A ubiquitous consensus exists regarding the ethical principle of truth-telling [27] that is the healthcare professionals have a duty to uphold patient autonomy and disclose errors that have a substantial impact on the patient’s health and well-being [28, 29]. It prompts healthcare professionals to promptly identify the errors in practice and explain the situation to the patient clearly and concisely, conveying any oral health issues and the potential consequences of inaction [30]. Additionally, offering a sincere apology is important, as it has been shown to increase patient compliance and decrease the likelihood of litigation [31]. A similar protocol was followed in our case, as the patient was promptly briefed about the incident and was offered an apology with reassurance.

Following the diagnosis and informing the patient, the subsequent step involves instrument retrieval. Several methods, categorized as surgical or non-surgical, exist for retrieving retained broken instrument segments [17] including tweezers or suction without specific manipulation, removal with another curette, or employing the double-ended magnetic instrument known as the Periotriever [17, 32]. In this particular case, a curved artery forceps was selected for its suitability for the intended task. Utilization of X-rays, MRIs, CT scans or metal detectors have been suggested in the literature to aid in the navigation of the broken fragment to minimize potential risk of damaging critical anatomical structures [33, 34]. Aperi-apical radiograph aided in accurately localizing the broken fragment in this case, which was then retrieved by curved artery forceps after creating a pathway by deflecting gingiva.

**Fig. 5:**
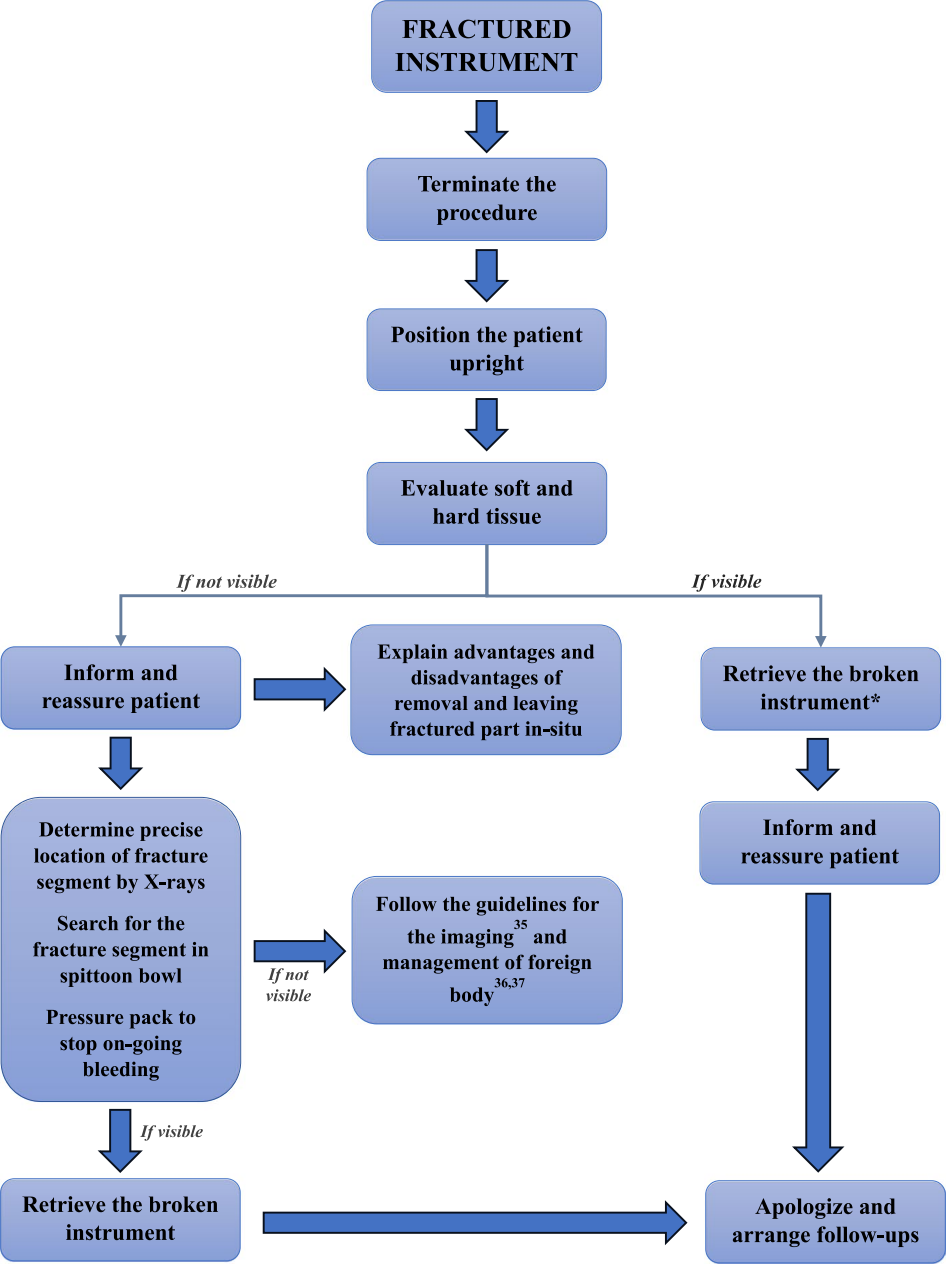
Protocol for instrument retrieval.  * Indicates that the fragment must be removed before informing when visible to prevent aspiration or ingestion

This case report reminds dentists about their ethical responsibilities and obligation to transparently inform patients about adverse events. The lack of established protocols highlighted the pressing need for guidelines. Based on the available evidence, the authors propose a protocol (Fig. 5) to manage such incidents with a published guideline for foreign body management [35–37]. It further encourages dental professionals to maintain composure and a calm demeanour during stressful situations, and signifies the importance of conducting thorough checks for instrument fatigue and ensuring correct equipment maintenance (Refer to Table 1 for recommendations regarding replacement of periodontal instruments).

**Table 1:** Recommendations regarding replacement of periodontal instruments

**Recommendation No.**	**Criteria for Replacement **[2, 3, 10, 38]
1.	Instruments displaying signs of wear, damage, and corrosion should be promptly replaced.
2.	Instruments should be replaced if the blade appears thin upon visual inspection. A blade width of 0.55 mm (approximately half of the initial width) serves as the criterion for discarding a curette or scaler.
3.	Significant wear, leading to tip distortion, becomes evident after 16 scaling cycles, necessitating the eventual disposal of the instrument.
4.	Retirement of an instrument is recommended when 20 percent of the blade width or length is reduced.
5.	Regular sharpening of instruments can result in a thin working end. Clinicians must observe and discard such instruments to prevent any mishaps.
6.	Unsharpenable instruments, once dulled, should be promptly replaced to maintain optimal performance.

## Key learning points

- Knowledge regarding instrument usage with proper technique and force.
- Honest and transparent communication by the clinician with the patient.
- Ensuring adequate maintenance of equipment and determining the appropriate time to dispose of instruments.
- Precisely determine the instrument’s location through radiographic examination rather than relying on a blind search.
- Maintaining calm and compose demeanour during challenging circumstances.

## Conclusion

Dental practitioners must exercise caution while using equipment in hard-to-reach regions, such as the periodontal pockets of posterior teeth. Routine and thorough instrument checks are necessary to ensure their integrity and functionality. It is crucial to have an in-depth understanding of the various types of instruments along with their techniques of usage. In instances of adverse events, as demonstrated in this case, adherence to ethical codes and guidelines is paramount. This involves promptly alerting the patient and appropriately managing the problem. Furthermore, consistent training for continual professional growth is essential to improve proficiency and minimize potential hazards during dental treatments.

## Data Availability

The datasets used and/or analysed during the current study available from the corresponding author on reasonable request.
